# Actigraphic Sensors Describe Stroke Severity in the Acute Phase: Implementing Multi-Parametric Monitoring in Stroke Unit

**DOI:** 10.3390/jcm12031178

**Published:** 2023-02-02

**Authors:** Giuseppe Reale, Chiara Iacovelli, Marco Rabuffetti, Paolo Manganotti, Lucio Marinelli, Simona Sacco, Giovanni Furlanis, Miloš Ajčević, Aurelia Zauli, Marco Moci, Silvia Giovannini, Simona Crosetti, Matteo Grazzini, Stefano Filippo Castiglia, Matteo Podestà, Paolo Calabresi, Maurizio Ferrarin, Pietro Caliandro

**Affiliations:** 1 UOC Neuroriabilitazione ad Alta Intensità, Dipartimento Neuroscienze, Organi di Senso, Torace, Fondazione Policlinico Universitario A. Gemelli IRCCS, 00168 Rome, Italy; 2 Department of Emergency, Anaesthesiology and Intensive Care Medicine, Fondazione Policlinico Universitario A. Gemelli IRCCS, 00168 Rome, Italy; 3 IRCCS Fondazione Don Carlo Gnocchi, 20148 Milan, Italy; 4 Clinical Unit of Neurology, Department of Medicine, Surgery and Health Sciences, Trieste University Hospital, University of Trieste, 34149 Trieste, Italy; 5 IRCCS Ospedale Policlinico San Martino, Department of Neuroscience, Division of Clinical Neurophysiology, 16132 Genova, Italy; 6 Department of Neuroscience, Rehabilitation, Ophthalmology, Genetics, Maternal and Child Health, University of Genoa, 16132 Genova, Italy; 7 Department of Biotechnological and Applied Clinical Sciences, University of L’Aquila, 67100 L’Aquila, Italy; 8 Department of Neuroscience, Catholic University of the Sacred Hearth, 00168 Rome, Italy; 9 Department of Medical and Surgical Sciences and Biotechnologies, “Sapienza” University of Rome-Polo Pontino, 04100 Latina, Italy; 10 Department of Brain and Behavioral Sciences, University of Pavia, 27100 Pavia, Italy; 11 UOC Neurologia, Dipartimento Neuroscienze, Organi di Senso, Torace, Fondazione Policlinico Universitario A. Gemelli IRCCS, 00168 Rome, Italy

**Keywords:** ischemic stroke, actigraphy, actigraphic parameters, actigraphic sensors, acute stroke, stroke unit

## Abstract

Actigraphy is a tool used to describe limb motor activity. Some actigraphic parameters, namely Motor Activity (MA) and Asymmetry Index (AR), correlate with stroke severity. However, a long-lasting actigraphic monitoring was never performed previously. We hypothesized that MA and AR can describe different clinical conditions during the evolution of the acute phase of stroke. We conducted a multicenter study and enrolled 69 stroke patients. NIHSS was assessed every hour and upper limbs’ motor activity was continuously recorded. We calculated MA and AR in the first hour after admission, after a significant clinical change (NIHSS ± 4) or at discharge. In a control group of 17 subjects, we calculated MA and AR normative values. We defined the best model to predict clinical status with multiple linear regression and identified actigraphic cut-off values to discriminate minor from major stroke (NIHSS ≥ 5) and NIHSS 5–9 from NIHSS ≥ 10. The AR cut-off value to discriminate between minor and major stroke (namely NIHSS ≥ 5) is 27% (sensitivity = 83%, specificity = 76% (AUC 0.86 *p* < 0.001), PPV = 89%, NPV = 42%). However, the combination of AR and MA of the non-paretic arm is the best model to predict NIHSS score (R^2^: 0.482, F: 54.13), discriminating minor from major stroke (sensitivity = 89%, specificity = 82%, PPV = 92%, NPV = 75%). The AR cut-off value of 53% identifies very severe stroke patients (NIHSS ≥ 10) (sensitivity = 82%, specificity = 74% (AUC 0.86 *p* < 0.001), PPV = 73%, NPV = 82%). Actigraphic parameters can reliably describe the overall severity of stroke patients with motor symptoms, supporting the addition of a wearable actigraphic system to the multi-parametric monitoring in stroke units.

## 1. Introduction

Stroke is a major cause of death and severe disability worldwide [1]. The acute phase is often dynamic and unstable since the clinical condition can change suddenly and dramatically. Therefore, it is important to admit patients into highly specialized stroke units that provide a continuous multi-parametric monitoring of blood pressure, oxygen saturation and ECG [2]. Ischemic stroke usually causes hemiparesis, which represents the most common residual disability [3]. The National Institute of Health Stroke Scale (NIHSS) is a widely used clinical scale to quantify the stroke severity and it is a strong outcome predictor both in the acute and subacute phase [4,5]. Motor symptoms have a major impact on the NIHSS final score (16 points out of 42). Therefore, tracking and measuring motor deficits could help to establish clinical severity, monitor symptoms evolution (or involution) and formulate a reliable prognosis [6]. In this context, actigraphy offers the chance to detect motor activity changes using wearable sensors placed on the patient’s wrists. In particular, Rabuffetti et al. developed among healthy individuals a numerical index able to quantify the motor activity of the upper limbs and to establish the asymmetry between the motor activity of the arms [7]. In a subsequent study conducted on a population of acute ischemic stroke patients, Iacovelli et al. [8] demonstrated that the asymmetry index, obtained by measuring upper limbs’ motor activity during a period of 24 h, was able to identify the paretic limb. Moreover, this index was found to correlate with the neurological condition of the patients, in terms of global stroke severity, measured with NIHSS total score, and paretic upper limb NIHSS motor sub-score.

Additionally, other recent studies [6] showed that the aforementioned asymmetry index is able to predict with good accuracy a 90 days severe prognosis (mRS > 2) and to distinguish motor deficit from motor neglect [9].

The above-cited studies took into consideration the actigraphic parameters measured within a 24 h window. However, a long-lasting monitoring of acute stroke patients through wearable devices might get a deeper insight into clinical evolution. In this frame, we hypothesized that the asymmetry index can be useful to describe different clinical conditions during the evolution of the acute phase of stroke, throughout the patient’s entire stay in the sub-intensive stroke unit.

The aims of this study were (1) to identify the actigraphy-based kinematic parameters that better characterize clinical severity of acute ischemic stroke patients; (2) to establish a cut-off value that helps to distinguish minor from major stroke, defined as NIHSS < 5 and NIHSS ≥ 5, respectively; (3) to establish a cut-off value able to identify very severe stroke patients (NIHSS ≥ 10).

## 2. Materials and Methods

### 2.1. Patients

We conducted a multicenter prospective observational study and enrolled consecutive patients with acute ischemic stroke during their hospitalization in a sub-intensive stroke unit.

The inclusion criteria were (a) ischemic stroke in the territory of the middle cerebral artery in the previous 72 h, regardless of the side, location and extension of the ischemic lesion or the clinical severity; (b) occurrence of hemiparesis or hemiplegia. The exclusion criteria were (a) previous stroke; (b) hemorrhagic stroke; (c) diagnosis of epilepsy and/or previous cognitive impairment; (d) anamnestic and/or instrumental evidence of previous upper limb motor impairment.

A neurologist specialized in cerebrovascular diseases assessed NIHSS at each patient’s admission into the stroke unit (NIHSS_T0_) and then every hour in order to detect any significant clinical change (defined as a change of at least 4 points in the NIHSS scale) [10]. During the night, NIHSS was assessed every hour in patients with severe stroke (NIHSS ≥ 10) and/or unstable hemodynamic or respiratory parameters. During the hospitalization, the motor activity of both arms was recorded using an accelerometer-based actigraphic system positioned on each wrist. Moreover, we collected clinical data including risk factors, reperfusion therapy, stroke localization and etiology.

The research was approved by the local ethics committee (Fondazione Policlinico Universitario A. Gemelli, Prot N. 0007987/17) and complies with the Helsinki Declaration. Informed written consent was obtained from each subject. One author had full access to all the data and took responsibility for their integrity and for data analysis.

### 2.2. Actigraphy

We continuously recorded the spontaneous upper limb movements using a wearable actigraphic system (GENEActiv; Activinsights, Kimbolton, Cambridgeshire, UK) consisting of a triaxial accelerometer integrated inside a wristwatch-like case, bilaterally positioned on the patients’ wrists. Actigraphic recordings started at the admission into the stroke unit department, after reperfusion therapies, when administered (thrombolysis and/or mechanical thrombectomy) and then lasted throughout all the patient’s hospitalization. Actigraphic recordings stopped during magnetic resonance performing. No patient refused to wear the actigraphic system. The two devices (one for each wrist) were synchronized in order to obtain the recordings of both limbs at the same time. The GENEactiv™ measured the wrist movement acceleration in three orthogonal directions at 100 Hz sampling rate. In agreement with Iacovelli et al. [8], we calculated the Motor Activity (MA) index for both the paretic and non-paretic arm, and then we obtained the Asymmetry Index (AR) between the two limbs.

Briefly, MA is an index of motor performance of upper limbs calculated as the norm value of the standard deviations of the acceleration components in the three axes according to the following equation, where $\sigma_{a_{x}}^{2},{\;\sigma }_{a_{y}}^{2}{\;\mathrm{and}\;\sigma }_{a_{z}}^{2}\;$ are the standard deviations of the acceleration components in the three axes [7,8]:

$$\mathrm{MA}=\sqrt{\sigma_{a_{x}}^{2}+\sigma_{a_{y}}^{2}+\sigma_{a_{z}}^{2}}$$

The AR index quantifies the asymmetry of movement between upper limbs as percentage values. When AR = 0% upper limbs’ motor activity is symmetrical, whereas a prevalence of right-side motor activity (up to a maximum of 100% if left activity is absent) is represented by positive values of AR and a prevalence of left-side motor activity (up to −100% if right arm activity is absent) is represented by negative values of AR. Therefore, left hemiparesis/hemiplegia is represented by positive values, while right hemiparesis/hemiplegia is represented by negative values of AR. It is noteworthy that nurses reported on a dedicated diary every moment in which the patient was passively mobilized and the actigraphic parameters were calculated after having removed those confounding intervals.

We calculated the mean values of MA and AR in the first hour after patient’s admission to the stroke unit every subsequent hour. In order to verify whether actigraphic measurements were able to detect conditions of different clinical severity, we considered for our statistical purpose the mean values of MA and AR in the first hour after admission and in the first hour following a significant clinical change (defined as a change of at least 4 points in the NIHSS scale). If such clinical change did not occur, we measured the actigraphic parameters and the NIHSS score at the end of hospitalization (NIHSS_T1_).

Moreover, we recruited a control group of 17 subjects (mean age 70.4 years SD 4.8, 7 males and 10 females) who were hospitalized and bed-restrained for orthopedic diseases not involving upper limbs and without any neurological symptoms. We chose a bed-restricted sample to replicate the setting of a stroke unit where patients are bed-restricted; this is also similar to the case of a minor stroke because of the continuous multi-parametric monitoring. Among them, we measured the MA of both arms for 24 h and then we calculated the AR, thus obtaining normative values for both the actigraphic parameters (AR mean value was 9.9% ± 7.0 and MA mean value was 194.7 (10^−3^ m/s^2^) ± 62.9).

Both indices (MA and AR) were calculated using MATLAB (The Mathworks, Natick, MA, USA). It is noteworthy that nurses reported on a dedicated diary every moment in which the patient was passively mobilized and the asymmetry index was calculated only after removing those confounding intervals in the analysis of MA index.

### 2.3. Statistics

All statistical analyses were performed in SPSS statistics software (version 20.0). MedCalc software was used for the sample size calculation. Based on a 90% prevalence of subjects with NIHSS ≥ 5 in the general population suffering from ischemic stroke [11], a required sample of at least 64 stroke patients was calculated to identify the actigraphic parameters with good ability to discriminate subjects with mild stroke from moderate-to-severe stroke (area under the receiver operating characteristic curve (AUC) ≥ 0.80) at 95% significance level and 80% power, under null hypothesis of AUC ≤ 0.50.

We used the Shapiro–Wilk probability test to assess the normality of the distributions. The Wilcoxon non-parametric test was used to compare NIHSS scores and sub-scores as well as the values of MA and AR indices between T0 and T1. Moreover, the same test was used to compare the values of MA between the upper limbs both at T0 and T1. The Spearman’s test was used to correlate MA and AR with NIHSS scores and upper limb motor sub scores at T0 and T1.

We used the multiple linear regression model with the stepwise method to identify the actigraphic parameters that best described the clinical severity. We considered NIHSS values as dependent variables, AR and MA as independent variables and adjusted the model for age and sex. We performed three different multivariate regressions in which the dependent and independent variables respectively were variables measured at T0, variables measured at T1 and variables measured at both T0 and T1. Once we identified the best actigraphic parameters to describe the NIHSS in the multiple linear regression model, we calculated the Receiver Operating Characteristic (ROC) curve in order to find the best actigraphic cut-off value able to discriminate minor (NIHSS < 5) from major stroke (NIHSS ≥ 5) and to identify very severe stroke patients (NIHSS ≥ 10). Specifically, we chose the kinematic value corresponding to the point of the ROC curve that maximized the sum of sensitivity and specificity. Moreover, we calculated both positive and negative predictive values (PPV and NPV, respectively) of the optimal cut-off point. *p* < 0.05 was set as the level of significance.

## 3. Results

### 3.1. Clinical Assessment

We recruited a total of 69 patients, but 5 patients were not considered in the following analysis because of low quality of actigraphic recordings, so a total of 64 patients were analyzed (36 females, median age 74 years range 31–94, 3 patients died). The overall hospitalization had a median duration of 4.83 days (range 3–10 days) and the median number of NIHSS/actigraphy measurements was 116 (range 21–168). Table 1 shows clinical characteristics of the enrolled patients as routinely collected in the setting of a stroke unit.

**Figure 1:**
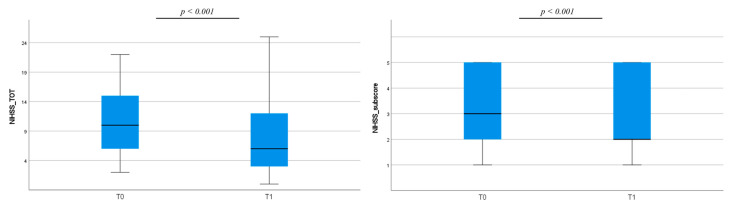
Clinical changes between T0 and T1 as measured by NIHSS total score (on the left). and NIHSS motor sub-score related to the paretic arm (on the right). NIHSS = National Institutes of Health Stroke Scale.

Clinical severity globally improved in the enrolled sample both in terms of NIHSS total scores and NIHSS motor sub-score of the paretic arm (Figure 1).

### 3.2. Actigraphic Assessment

**Figure 2:**
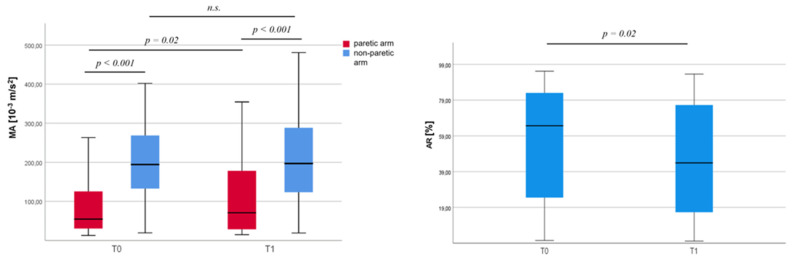
Changes of actigraphic parameters between T0 and T1. AR = Asymmetry Index, MA = Motor Activity index.

Spontaneous motor activity of the paretic arm was lower than that of the non-paretic one at both T0 and T1, and it increased (namely, it improved) between the two time points (Figure 2A, while motor activity of the non-paretic arm did not change over time (Figure 2A). Consequently, the motor activity profiles of the arms were more symmetric at T1 than at T0 as the AR variation demonstrated, specifically, AR absolute values were lower at T1 than at T0 (Figure 2B).

**Figure 3:**
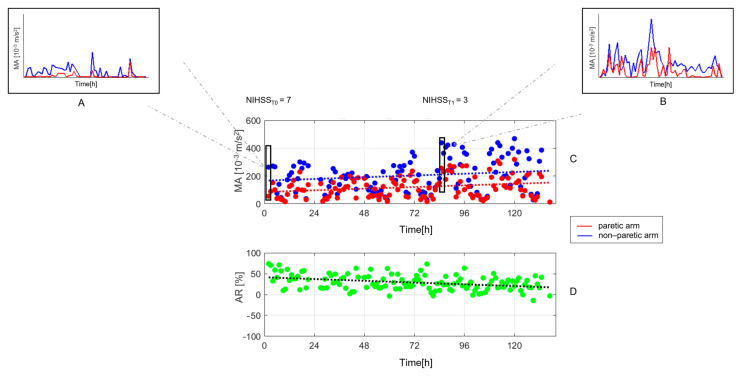
Recordings of actigraphic parameters during the stroke unit stay in a patient whose clinical severity improves over time. The upper boxes (**A**,**B**) show motor profiles of paretic (red line) and non-paretic arm (blue line) at T0 and T1. Boxes (**C**,**D**), respectively, show one-hour mean values of MA and AR. NIHSS = National Institutes of Health Stroke Scale, AR = Asymmetry Index, MA = Motor Activity index.

Figure 3 shows the actigraphic recordings of a patient whose clinical severity improves from T0 to T1.

**Figure 4:**
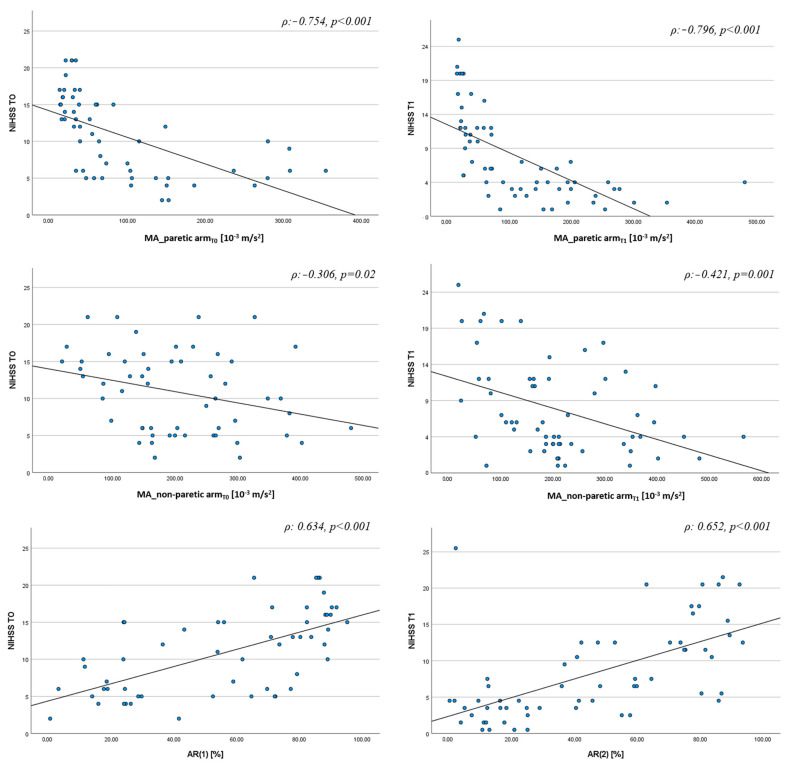
Correlation analysis between actigraphic indices and NIHSS scores at T0 and T1. NIHSS = National Institutes of Health Stroke Scale, AR = Asymmetry Index, MA = Motor Activity index.

Univariate analysis showed a negative correlation between MA of the paretic arm and NIHSS score at T0 (ρ: −0.754 *p* < 0.001) and at T1 (ρ: −0.796 *p* < 0.001), as well as between MA of the non-paretic arm and NIHSS score at T0 (ρ: −0.306 *p* = 0.02) and T1 (ρ: −0.421 *p* = 0.001). We found a positive correlation between AR and NIHSS score both at T0 (ρ: 0.634 *p* < 0.001) and T1 (ρ: 0.652 *p* < 0.001) (Figure 4). We found similar results for the correlation between MA of the paretic arm and NIHSS sub-score of the paretic arm at T0 (ρ: −0.748 *p* < 0.001) and T1 (ρ: −0.785 *p* < 0.001) as well as between AR and NIHSS sub-score of the paretic arm at T0 (ρ: 0.622 *p* < 0.001) and T1 (ρ: 0.720 *p* < 0.001).

From multivariate analysis (Table 2), we found that the best independent predictors of NIHSS total score at T0 were AR and MA of the non-paretic arm, while at T1 the best predictors were AR and MA of the paretic arm. A third multivariate model demonstrated that the association of AR and MA of the non-paretic arm properly predicted NIHSS score regardless of the timing of assessment of NIHSS and actigraphic parameters.

**Figure 5:**
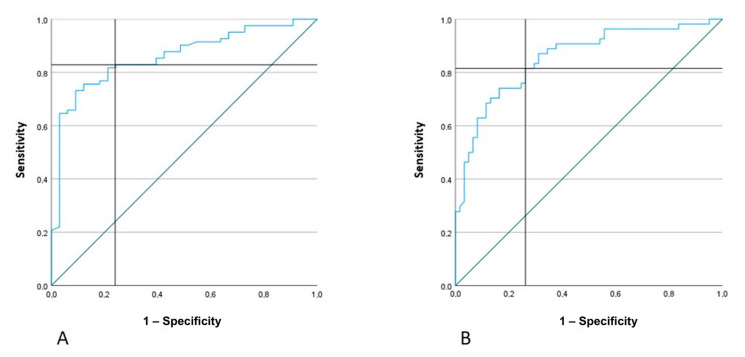
ROC curves to find the best AR cut-off values able to discriminate, respectively, NIHSS ≥ 5 (**A**) and NIHSS ≥ 10 (**B**). AR = Asymmetry Index, NIHSS = National Institutes of Health Stroke Scale.

Since the regression models demonstrate that AR was the main predictor of clinical severity, after building ROC curves, we established that a cut-off value of 27% for AR (Figure 5A) was able to discriminate between minor and major stroke (NIHSS ≥ 5). This means that an AR index of 27% or higher corresponds to a NIHSS ≥ 5 with a sensitivity of 83% and a specificity of 76%, and the Area Under Curve (AUC) = 0.86, with *p* < 0.001. The PPV was 89% and the NPV 42%. The NPV of this cut-off value is actually low. In other words, an AR value lower than 27%, which represents a substantial motor symmetry between the arms, could actually identify as minor stroke also patients with a severe clinical condition (namely NIHSS ≥ 5). According to our regression model results though, the combination of AR and MA of the non-paretic arm is the best model to describe the clinical picture, regardless of the timing of the clinical evaluation. Therefore, we considered these two parameters together in order to improve the NPV value and the ability to discriminate between minor (NIHSS < 5) and major stroke (NIHSS ≥ 5). We identified a value of 130 (10^−3^ m/s^2^) for MA as the lowest limit of motor activity in the control group, meaning that values ≥130 (10^−3^ m/s^2^) indicate unimpaired motor skills. Therefore, whenever the AR was lower than 27%, we also took into consideration the corresponding value of MA of the non-paretic arm to better identify each patient as having minor or major stroke, adopting the following algorithm:

1. if AR < 27% and MA of the non-paretic arm <130, the patient has NIHSS ≥ 5
2. if AR < 27% and MA of the non-paretic arm >130, the patient has NIHSS < 5
3. if AR > 27%, regardless of MA value, the patient has NIHSS ≥ 5

In this way, after combining the two actigraphic parameters (AR and MA), we obtained the following results: sensitivity = 89%, specificity = 82%, PPV = 92% and NPV = 75%.

Moreover, we obtained the ROC curve for the asymmetry index (Figure 5B) to identify the best cut-off value able to identify very severe stroke patients (NIHSS ≥ 10). The AR value of 53% presented the following results: sensitivity = 82% specificity = 74% (AUC 0.86 *p* < 0.001) PPV = 73% NPP = 82%.

## 4. Discussion

We were able to identify the actigraphic parameters that best predict clinical severity during the acute stroke phase. Moreover, after combining them, we were able to find reliable cut-off values that, put together, can distinguish minor from major stroke with acceptable sensitivity (89%) and specificity (82%). These findings suggest that continuous actigraphic monitoring of upper limbs’ motor activity could usefully implement the already-existing multi-parametric monitoring in the setting of a stroke unit. In fact, clinical fluctuations are very common in the first days after an ischemic event and it could be hard to detect them promptly through serial clinical observations. A fast detection of a clinical worsening could be decisive in the optimal management of patients with unstable underlying conditions, such as extra- or intracranial arterial stenosis, or those at risk of malignant cerebral edema or hemorrhagic transformation. Currently, there is no reliable technology able to identify significant changes of the clinical status. Actigraphy has been extensively used in previous studies to assess motor performance of stroke patients [9,12,13].

Excluding a vast literature on actigraphic recordings of outpatients under everyday-living conditions, studies conducted on acute stroke patients mainly focused on upper limb performance, evaluated with many clinical outcome measures such as NIHSS upper limb sub-score, ARAT, Fugl–Meyer and other scales [14,15,16]. Moreover, recordings were generally performed days after the acute stroke and continuous recordings were usually shorter than 48–72 h [15]. Conversely, our study focused on patients in the hyper-acute phase and included a longer monitoring during all the hospitalization in the stroke unit department. Moreover, our aim was not only to assess upper limb motor performance, but also to derive an estimate of the overall clinical severity from actigraphic parameters. In fact, in previous studies, we demonstrated that actigraphic parameters were able to identify paretic limbs with high accuracy and correlated with clinical severity (NIHSS) [8] and 90 days prognosis (mRS) [6]. In this frame, the data from this study, obtained using wristwatch-like sensors in the setting of a stroke unit, show that actigraphic parameters (asymmetry index and motor activity) are able to promptly detect clinical changes. In particular, we found a positive correlation between the AR and the NIHSS both at T0 and T1, meaning that a higher value of this index corresponds to a more severe clinical condition as evaluated with NIHSS. We also established a cut-off value of this index (AR > 27%) able to properly discriminate between minor and major stroke with a high PPV (92%). Therefore, we can state with adequate certainty that an AR value higher than 27% corresponds to a patient having a NIHSS ≥ 5 (major stroke). However, the NPV of this cut-off value is low. In other words, an AR value lower than 27% identifies as having minor stroke also patients with a severe clinical status (namely NIHSS ≥ 5). This might depend on the fact that patients with severe neurological conditions, such as major stroke, often have reduced consciousness, with a consequent reduction of the motor activity of both arms. Therefore, these patients have a high symmetry between the MA of both arms and an AR value often lower than 27%, despite being in a very severe clinical condition. This issue could be overcome putting together both the asymmetry index and the motor activity. Specifically, in order to understand whether such a low value of AR refers to a better or worse clinical condition, we combined it with the MA actigraphic parameter. As previously stated, we established a range of normative values for MA in a control group of bed-restrained patients not experiencing neurological symptoms, i.e., without motor impairment of upper limbs, 130 being the lowest possible MA value in this group. Therefore, a combination of an AR value lower than 27% and a MA value <130 of the non-paretic arm in a stroke patient is strongly suggestive of a severe clinical condition (NIHSS ≥ 5). On the other hand, a combination of an AR value lower than 27% and a MA value higher than 130 correctly identifies a subject with minor stroke (NIHSS 0–4). In this way, we obtained both a high PPV value (92%) and a high NPV value (75%). Therefore, the actigraphic system can reliably describe the overall severity of stroke patients with motor symptoms.

Notably, the population we recruited only included patients with ischemic stroke in the middle cerebral artery territory and experiencing hemiparesis. Therefore, we do not know if the selected actigraphic parameters are also informative in posterior stroke patients and in patients with ischemic stroke in the territory of middle cerebral artery but without motor impairment.

## 5. Conclusions

In conclusion, our data support the addition of a wearable actigraphic system that continuously records upper limbs’ motor activity to the standard multi-parametric monitoring in stroke unit.

## Figures and Tables

**Table 1:** Clinical and demographic data of the enrolled patients.

Clinical Data	Frequency (%)
**Risk factors**	
Atrial Fibrillation	21 (32.8%)
Arterial Hypertension	40 (62.5%)
Dyslipidemia	19 (29.7)
Smoke	12 (18.7%)
Diabetes Mellitus	8 (12.5%)
**Clinical features**	
Left Hemisphere	31 (48.4%)
NIHSS < 5 at T0 NIHSS 5–9 at T0 NIHSS ≥ 10 at T0	6 (9.3%) 21 (32.8%) 37 (57.8%)
NIHSS < 5 at T1 NIHSS 5–9 at T1 NIHSS ≥ 10 at T1	27 (44%) 12 (20%) 22 (36%)
**Reperfusion therapies** Thrombolysis Mechanical thrombectomy Thrombolysis and Mechanical thrombectomy **Ischemic lesion**	10 (16%) 9 (14%) 6 (10%)
Superficial	17 (26.5%)
Deep	20 (31.2%)
Superficial and Deep	27 (42.2%)
**Etiology (TOAST)**	
Large artery atherosclerosis	11 (17.2%)
Small vessel disease	16 (25%)
Cardioembolic	15 (23.4%)
Other causes	4 (6.2%)
Undetermined	18 (28.1%)

NIHSS = National Institutes of Health Stroke Scale; TOAST=Trial of Org 10172 in Acute Stroke Treatment.

**Table 2:** Stepwise Multiple Linear Regression Analysis. NIHSST0, NIHSST1 and NIHSST0/T1 as Dependent Variables. Beta Coefficient and P Value.

Variables	NIHSS T0	NIHSS T1	NIHSS T0/T1
AR	0.616 (<0.001)	0.312 (0.023)	0.603 (<0.001)
MA_non-paretic arm	−0.285 (0.007)	----	−0.314 (<0.001)
MA_paretic arm	----	−0.431 (0.002)	----
R^2^ F	0.446 22.76	0.450 25.10	0.482 54.13

NIHSS = National Institutes of Health Stroke Scale, AR = Asymmetry Index, MA = Motor Activity index.

## Data Availability

The datasets generated during and/or analyzed during the current study are available from the corresponding author on reasonable request.
